# Use of Phenytoin, Phenobarbital Carbamazepine, Levetiracetam Lamotrigine and Valproate in Pregnancy and Breastfeeding: Risk of Major Malformations, Dose-dependency, Monotherapy *vs* Polytherapy, Pharmacokinetics and Clinical Implications

**DOI:** 10.2174/1570159X19666210211150856

**Published:** 2021-02-11

**Authors:** Yusuf Cem Kaplan, Omer Demir

**Affiliations:** 1 Terafar - Izmir Katip Celebi University Teratology Information, Training and Research Center, Izmir, Turkey;; 2 Izmir Katip Celebi University School of Medicine, Department of Pharmacology Izmir, Turkey;; 3 Izmir University of Economics Faculty of Medicine, Izmir, Turkey;; 4 University of Health Sciences, Izmir Tepecik Training and Research Hospital, Teratology Information Center, Izmir, Turkey

**Keywords:** Pregnancy, breastfeeding, antiepileptics, major malformations, phenytoin, carbamazepine, phenobarbital, valproate, lamotrigine, levetiracetam

## Abstract

It is challenging to balance the fetal risks associated with the use of antiepileptic drugs (AEDs) against maternal and fetal risks of seizure worsening, and therefore it is very important to define and distinguish the possible risks entailed by different AEDs. This paper aims to undertake a comprehensive review regarding the possible risks of four classical (phenytoin, carbamazepine, phenobarbital, and valproate) and two newer (lamotrigine and levetiracetam) AEDs during pregnancy. The review focuses on major and organ-specific malformations, dose-dependent risks, mono *vs* polytherapy, and clinical pharmacokinetics. A discussion regarding the safety of AED use during breastfeeding is also provided.

## 1 INTRODUCTION

Antiepileptic drug (AED) therapy during pregnancy is extremely challenging both for pregnant women and their physicians. The well-known fetal adverse effects of AEDs should be balanced with the need for optimum seizure control. It is important to highlight that this challenge is not limited to women with epilepsy (WWE), since AEDs are used in the treatment of other chronic disorders, such as migraine, bipolar disorder, and neuropathic pain.

To date, significant research efforts in this domain have led to a myriad of studies aiming to delineate and distinguish the possible risks of maternal AED use on fetus. As a result of the ever-growing body of literature, knowledge of the distinct teratogenic effects of different AEDs has advanced greatly over the last decade, which enabled the clinicians to reduce the potential risks by modifying the type and the dose of the AEDs prior to the pregnancy, in order to achieve better maternal and fetal outcomes.

The aim of this review is to undertake a comparative evaluation regarding the possible risks of four conventional AEDs phenytoin (PHT), phenobarbital (PB), carbamazepine (CBZ) and valproate (VPA) use during pregnancy and breastfeeding, against two frequently used newer AEDs; lamotrigine (LTG) and levetiracetam (LEV) in the context of major and organ-specific malformations, dose-dependent risks, mono *vs* polytherapy, clinical pharmacokinetics and safety during breastfeeding. The neurodevelopmental context will be assessed in detail in another review in the same issue and is excluded here. Other adverse pregnancy outcomes are also excluded since the data are not sufficient to perform a comparison in terms of particular AEDs.

Although the findings from the different pregnancy registries for particular AEDs are presented together in the relevant sections, the reader should be aware that significant differences may exist between pregnancy registries; therefore a direct comparison of the free-standing risk rates would be problematic Table (**1**). As summarized by Tomson *et al*. [1], some examples of these disparities can be given as follows:

● Differences in the enrolment and follow-up methodologies exist. For the UK registry, half of the enrolled pregnancies are self-referrals, whereas the second half is referred to through health care personnel. The follow-up is made with a report at the 3rd month after birth. The North American Pregnancy Registry recruits pregnant women by self-referral by calling a toll-free number. There are two follow-ups, which are conducted by a telephone call at 7 months of pregnancy and 8-12 weeks after the estimated date of delivery. EURAP conducts the enrolment through a network of collaborating physicians in several countries. The follow-up is more detailed, which is done after each-trimester and at 1 year after the birth. These differences can introduce different types of biases. For the UK registry, the information gained is more limited than the other two. In the North American Pregnancy Registry, because the enrolment is conducted by a self-referral, a selection bias towards more aware and better-informed women can be present. For the EURAP, because the physicians refer the pregnant women for the enrolment, a bias towards more severe epilepsy may exist.

● The definition of “prospective enrolment” differs between registries. For instance, the North American Pregnancy Registry includes the women at any time during pregnancy and women are considered as “purely prospective” or “traditional prospective” depending on whether they were included too early or after 16-20 weeks (after prenatal screening and ultrasound). The UK registry and EURAP classify the pregnancies as prospective if they are enrolled before the outcome is known, however EURAP is considered to have stricter criteria since it includes the pregnancies which were referred before the 16th week of pregnancy. As a consequence, different types of biases, such as the selection and reporting biases for the “traditional prospective” pregnancies for the North American Pregnancy Registry, can be present in different registries.

● The registries also differ among their exposure classification. EURAP and the North American Pregnancy Registry exclude pregnancies in which the changes regarding the AED occur during the first trimester. However, the UK registry, for instance, classifies the pregnancies which changed from mono to polytherapy as polytherapy. In addition, if the AED is stopped during the first trimester, it is classified as the original treatment.

The registries may also vary regarding the malformation classification. For instance, Scheuerle *et al*. have demonstrated these substantial differences regarding case classification between EURAP and the North American Pregnancy Registry in their prospective LEV registry [2]. In EURAP, 22 of the 47 cases were classified as having a major malformation, whereas 7 of the same 47 cases were classified as having a major malformation in the North American Pregnancy Registry [2].

Although all these registries obtain information regarding the potential confounders, there are differences in some collected variables (smoking and alcohol), collection methods (healthcare personnel, phone, medical records or combinations), and definitions (family history), which may also lead to differences in the analyses.

It is important to note that the context of this paper reflects the perspective of the authors, who have worked as consulting physicians in the clinical teratology units for several years with a clinical pharmacology and toxicology background; *i.e.*, it represents only a part of the complex clinical decision-making process regarding the choice of an AED in a pregnant patient. Every decision regarding the pharmacotherapy in pregnant WWE should be individualized through the careful consideration of clinical factors (*e.g.* seizure type and severity, prior response to AEDs, comorbidities), and the evidence regarding AEDs (*e.g.* efficacy, safety *etc.*). Thus, it is crucial to building a mutual understanding between the patient and the physician regarding the teratogenic risk perception and their expectations from the treatment. When counseling a women of reproductive age, it is important to inform her about the baseline rate of malformations that exists for all healthy women [3]. The women should be explained that even without exposure to a drug or treatment, there is still a risk of having an infant with major malformations which is 1-3% [4]. If a range would not be preferred, than 2% as shown in two large, population-based, well-designed studies, can be used [5, 6]. It should always be remembered that framing can exert a significant influence on how the patient would perceive the possible risk and her decisions [7]. Nevertheless, the communication of the possible risks comprises significant difficulties and biases, and the ways to overcome these challenges are discussed elsewhere [7].

## 2 MAJOR CONGENITAL AND ORGAN-SPECIFIC MALFORMATIONS

### 2.1 Phenytoin

During 1970s, a pattern of malformations, which were referred to as fetal hydantoin syndrome, were associated with in utero phenytoin exposure in a series of cases. Common manifestations were abnormal facial and skull features, such as broad low nasal bridge, epicentral folds, a short upturned nose, low set ears, head size variations, wide fontanels and sutural ridging, as well as limb features such as hypoplasia of nails and distal phalanges, fingerlike thumbs, and variations in palmar creases [8]. There were also delays in growth, motor skills and mental development [8, 9]. The most consistent findings were considered to be craniofacial and limb features [10]; however, subsequent reports and studies raised some concerns regarding the definition of this syndrome [10, 11].

Studies to date reported different rates of major malformations following PHT exposure during pregnancy and cannot be considered as consistent in terms of a significant increase in major malformation risk. One of the earlier analyses of the Australian Antiepileptic Drugs Pregnancy Registry suggested a prevalence of 10.5% [12], whereas analysis of the same registry in 2007 and 2019 yielded much lower prevalences (5.9% and 2.3%, respectively) [13, 14]. However, the numbers of the total PHT exposed pregnancies in all these series were very low (n=17-44) [13]. A prospective analysis of 983 infants born in Japan, Italy, and Canada reported a 9.1% risk of congenital malformations with fetal PHT exposure, compared with 3.1% in those not exposed to AEDs [15].

No association between maternal PHT monotherapy and major malformations in the infants (n=124) was found in the study by Kaaja *et al*. [16]. The analysis of prospective UK Epilepsy and Pregnancy Register by Morrow *et al*. suggested no significant increase in the rate of major malformations in the infants of mothers who used PHT during pregnancy compared with those who used no AEDs (3.7% *vs* 3.5%) [17]. An evaluation of the North American AED Pregnancy Registry reported no excessive increase in risk (2.9%) of major malformations in pregnancies exposed to PHT monotherapy during the first trimester [18]. The prevalence of major malformations following PHT monotherapy was found as 6.4% in the recent analysis of EURAP registry data [19].

In their meta-analysis, Meador *et al*. reported a 7.36% risk of MCMs for phenytoin monotherapy compared with a rate of 3.27% in the control group (not exposed to AEDs), but the difference was not statistically significant due to large variance in the data [20]. Weston *et al*. in their comprehensive meta-analysis, reported the prevalence of the major malformations as 6.26% (95% CI 4.37 - 8.47) [21]. The authors reported that the variation regarding the prevalence among the studies was significant. In the comparisons, children exposed to PHT monotherapy in utero were reported to have a significant increase in major malformation risk when compared to those of women without epilepsy (RR 2.38; 95% CI 1.12 - 5.03), and those of the women with epilepsy who used no medications (RR 2.40; 95% CI 1.42 - 4.08). They found no significant associations between PHT and organ specific malformations, but pointed out the limitations of the available data in this context [21]. The results from a recent network meta-analysis by Veroniki *et al*. were also in line with the previous meta-analyses [22]. They reported a significant OR of 1.67 (95% CI 1.30 - 2.17) for major malformations in the infants following maternal PHT therapy during pregnancy. In addition, PHT monotherapy was also significantly associated with cleft lip/palate (OR, 3.11; 95% CI 1.31– 7.72), and club foot (OR, 2.73; 95% CI 1.13–6.18). As monotherapy, no significant associations were evident for cardiac malformations, hypospadias, and inguinal hernia [22]. However, as Tomson *et al*. pointed out, heterogeneity of the data extracted from the included studies, particularly in terms of the outcome criteria and time window for their detection is an important limitation in meta-analysis [23]. The definition of the major congenital malformations can vary across studies, and this may lead to inclusion of less severe anomalies as major malformations in the pooled data, which may be a limitation for the meta-analyses [23].

### 2.2 Phenobarbital

The prevalences of major malformations were more consistent among the studies investigating maternal PB exposure. In the study by Kaneko *et al*. the major malformation prevalence was 5.1% among the children who had in utero PB exposure [15], while the North American AED Pregnancy Registry reported an MCM prevalence of 5.5% [18]. In the most recent analysis of the EURAP registry, Tomson *et al*., reported a similar yet slightly higher major malformation prevalence for PB, which was 6.5% [19]. Using the French National Health Insurance data, Blotière *et al*. investigated the association of 23 specific malformations following exposure to AEDs during the first two months of pregnancy. The authors detected a significantly increased risk for ventricular septal defect following PB use (OR 10.5; 95% CI 1.3– 39.3), but no significant associations with other specific malformations [24].

In their meta-analysis, Weston *et al*. reported the prevalence of major malformations as 7.10% (95% CI 5.36 -9.08) following maternal PB exposure [21]. Further comparisons with the children of women without epilepsy (RR 2.84; 95% CI 1.57 - 5.13), and children of those with epilepsy who used no medications (RR 1.95; 95% CI 0.97 - 3.93) yielded different results where the former showed a significant increase in risk. Furthermore, organ-specific malformation analysis suggested no significant increases in risk for both comparison groups. In the meta-analysis by Veroniki *et al*., a significant increase in the risk of major congenital malformations with PB exposure (OR 1.83; 95% CI 1.35–2.47) was detected. Furthermore, PB monotherapy was significantly associated with an increased risk of cleft lip/palate (OR 5.75; 95% CI 2.41– 14.08). When given as a monotherapy, no significant associations were reported for cardiac malformations, hypospadias, club foot or inguinal hernia [22].

### 2.3 Carbamazepine

The analysis of the Swedish Birth registry in 2004 revealed a major malformation rate of 3.9% (classified as “severe” malformations n=28/703) following CBZ exposure during pregnancy [25].

An earlier analysis of the North American Pregnancy Registry reported no significant increase in the risk of major malformations for infants (2.6%) exposed in utero to CBZ monotherapy when compared with the major malformation rate in the general population [26]. Similar findings emerged from a more recent analysis of the same registry; the prevalence of major malformations with CBZ exposure was 3.0%, and the risk ratio was not significantly higher than controls [18]. In addition, the significant increase in risk for cleft lip/palate and neural tube defect reported by the earlier study (Holmes and Wyszynski, 2004) was not evident in the latter using the same registry data [18]. The analysis of the Health Improvement Network (THIN) cohort by Ban *et al*. in 2015 yielded a prevalence of 4.2% for any major malformations following CBZ monotherapy during the first trimester of pregnancy. This rate was not significantly higher than the major malformation rate in those without AED exposure in the first trimester (OR 1.58; 95% CI 0.86–2.89) [27]. In the EURAP registry, the prevalence of major malformations following CBZ use during pregnancy was 5.5% [19]. The recent analysis of the Australian Pregnancy Register detected a prevalence of 5.9% for major malformations with CBZ monotherapy during pregnancy. The comparison of this rate with that of untreated women suggested a trend towards an increased risk for major malformations, although the RR (2.07; 95% CI 0.80 - 5.33) was not statistically significant [14]. Another recent analysis of the French National Health Insurance data focusing on the association of AED use with 23 specific malformations found no excess risk for any of these following CBZ use during the first two months of pregnancy [24].

Jentink *et al*. used the EUROCAT database to assess the signals of the specific congenital malformations (spina bifida, cleft lip, diaphragmatic hernia, hypospadias while total pulmonary venous return) detected during their systematic review of cohort studies regarding CBZ exposure during pregnancy [28]. Based on their investigation of eight eligible cohorts, the pooled prevalence for a major malformation following CBZ monotherapy during pregnancy was 3.3% [28]. Of importance, this value is lower than that was reported in an earlier meta-analysis as 5.3% by Matalon *et al*. [29]. Apart from the spina bifida, discussed in the sub-section below, the authors detected no significant increases in risk for cleft lip (with or without palate), diaphragmatic hernia, or hypospadias. There were no cases of total pulmonary venous return to investigate [29].

There are three notable meta-analyses for CBZ exposure and major malformations. The earliest, by Matalon *et al*., reported a significantly increased risk for major malformations in children born from the mothers receiving CBZ monotherapy during their pregnancies (OR 2.45; 95% CI 1.66 - 3.62). Based on 30 studies, Weston *et al*. found the pooled prevalence of major malformations in children exposed to CBZ as 3.71% [21], only slightly above the figure of 3.3% reported by Jentink *et al*. [28]. The comparisons revealed a significant increase in the risk of major malformations among the group of children exposed to CBZ in-utero when compared with the children of mothers without epilepsy (RR 2.01; 95% CI 1.20 - 3.36) and the children of epileptic mothers using no medication during pregnancies (RR 1.50; 95% CI 1.03 - 2.19). The risk ratios were not significant for neural tube and cardiac malformations in both comparison groups. However, orofacial cleft/craniofacial malformations analyses yielded significant increases in risk when CBZ-exposed children were compared with those of women without epilepsy (RR 6.13; 95% CI 1.19 - 31.49). Nevertheless, the risk to CBZ-exposed children was not significant when compared to the children of epileptic mothers with no AED use during pregnancy [21]. Findings from the meta-analysis by Veroniki *et al*. were comparable with the previous meta-analyses, since an increased risk of major malformations with CBZ monotherapy during pregnancy was reported (OR 1.37; 95% CI 1.10 - 1.71). Nevertheless, no significant increase in risk was apparent for organ-specific malformations, such as cardiac malformations and cleft/lip palate [22].

#### 2.3.1 Carbamazepine and Spina Bifida

Reports during 1980s implied an association of spina bifida among infants with in utero exposure to CBZ monotherapy, as well as polytherapy with other AED [30]. Subsequent cohort studies demonstrated that CBZ monotherapy during pregnancy is associated with a 2- to 10-fold increase in the risk of spina bifida. For instance, in the case-control study by Jentink *et al*., the authors reported a significant increase in the risk of spina bifida when compared to unexposed controls (OR 2.6; 95% CI 1.2–5.3) [28]. Although a 2- to 10-fold increase in odds may seem high, it approximates a relatively small absolute risk of between 0.2 and 1%, considering the low prevalence of this malformation (1:1000) [31-33].

### 2.4 Valproate

As a result of studies during the last two decades, VPA emerged as a teratogen, which may increase the risk of overall and organ-specific malformations, such as neural tube defects, cardiac and limb anomalies.

First reports pointing to the teratogenic effects of VPA in humans, particularly spina bifida, began to appear in the early 1980s [34, 35]. DiLiberti *et al*. and other authors suggested that VPA use during pregnancy might cause a specific “VPA syndrome” in the offspring, characterized by consistent facial features such as flat nasal bridge, epicanthal folds, long upper lip and thin vermillion border, as well as some neurodevelopmental abnormalities [36-39]. However, subsequent studies raised doubts since the suggested syndrome did not appear to be different than “antiepileptic drug syndrome” [35, 40]. During 1990s and 2000s, prospective observational studies consistently reported a significantly increased rate of major malformations in infants whose mothers used VPA during pregnancy. Of those available, the following studies made a notable impact within this domain of research. In their multicenter study including data from five European centers, Samrén *et al*. reported a rate of 9% for major malformations in children following in utero exposure to VPA [41]. Kaneko *et al*. in their collaborative study, based on prospectively collected data from Japan, Italy and Canada, detected a major malformation incidence of 11.1% in infants whose mothers were administered VPA monotherapy, significantly higher than those without drug exposure (3.1%) [15]. In the analysis of the Finnish Birth Registry by Artama *et al*., VPA monotherapy during pregnancy was found to significantly increase the risk of major malformations (OR 4.18; 95% CI 2.31–7.57) when compared with those using no AED during pregnancy [42]. Morrow *et al*. assessed the prospective data collected by UK Epilepsy and Pregnancy Register and reported the major malformation rate in those taking VPA during pregnancy as 6.2% (95% CI, 4.6% to 8.2%, adjusted OR 2.97; 95% CI 1.65–5.35 in comparison to CBZ); the major malformation rate in women using CBZ was 2.2% (1.4% to 3.4%), and in women with epilepsy not using AEDs during pregnancy, 3.5% (1.8% to 6.8%) [17]. Meador *et al*. reported a much higher rate, (17.4%) in their prospective cohort study pooling data from the 25 epilepsy centers from the U.S. and UK regarding major malformations following VPA monotherapy [43]. A cohort study by Diav-Citrin *et al*. reported the rate of major anomalies in VPA-exposed pregnancies as 6.7%, which corresponded to a significantly elevated relative risk of 2.66 (95% CI 1.25 - 5.65) when compared with the pregnancies exposed to non-teratogenic medications [44].

The analysis of EUROCAT database in 2010 by Jentink *et al*. suggested several associations between VPA exposure in the first trimester and organ-specific malformations when compared with pregnancies with no exposure to AEDs [28]. In their case-control study, the authors detected that the exposed infants had greater risks of between 2 to 7 times for atrial septal defect, cleft palate, craniosynostosis, hypospadias, polydactyly, while the risk for spina bifida was 12 to 16 times higher depending on the control group. This finding was comparable with the literature. In the same study, the authors also conducted a review of the cohort studies and identified 14 malformations, including the six mentioned above, that were significantly associated with VPA monotherapy during the first trimester [28].

Hernandez-Diaz *et al*. reported a rate of 9.3% major malformations following gestational VPA monotherapy in North American AED Pregnancy Registry. The relative risk was approximately 5-fold higher when compared to that of lamotrigine (RR 5.1; 95% CI 3.0-8.5) [18]. VPA exposure was also found to be associated with an increased risk of cardiovascular malformations, hypospadias and neural tube defects [18]. In the latest analysis of the Australian Register of Antiepileptic Drugs in Pregnancy in 2019, Vajda *et al*. detected a prevalence of 14.8% for the risk of major malformations following in utero exposure to VPA monotherapy. This value corresponded to a significant risk ratio of 5.22 (95% CI 3.0-8.5) when compared with the fetuses AED-unexposed pregnancies [14]. Tomson *et al*. reported a slightly lower malformation prevalence of 10.3% in the recent analysis of EURAP registry [19].

The 2006 meta-analysis of Koren *et al*. revealed a significant 2.59-fold (95% CI 2.11-3.17) increase in the risk of major malformations among the infants of mothers using VPA monotherapy during pregnancy when compared with those using other AED monotherapies [45]. The relative risk was found to be further increased when untreated epileptic mothers (RR 3.16; 95% CI 2.17-4.60) or healthy mothers (RR 3.77; 95% CI 2.18-6.52) were taken as controls [45]. Another earlier meta-analysis by Meador *et al*. reported a pooled incidence of 10.73% (95% CI 8.16-13.29), the highest rate among all the AEDs included, for major malformations following in utero exposure to VPA monotherapy [20], a finding reflected in recent meta-analyses. Weston *et al*. calculated a pooled prevalence of 10.93% for any type of major malformation in children exposed to VPA [21]. The risk ratio was 5.69 (95% CI 3.33-9.73) when VPA-exposed children were compared with those of healthy women taking no drugs during pregnancy, and 3.13 (95% CI 2.16-4.54) when VPA-exposed children were compared with those of epileptic women taking no drugs during pregnancy [21]. The authors also detected significantly elevated risk ratios for neural tube defects (~ 5 fold), cardiac malformations (4 to 16 fold) and orofacial clefts (~ 5 fold) [21]. The meta-analysis by Veroniki *et al*. showed increases in the risk of major malformations (OR 2.93; 95% CI 2.36-3.69), hypospadias (OR 2.58; 95% CI 1.24-5.76), cleft lip/palate (OR 3.26; 95% CI 1.38-5.58) and club foot (OR 3.26; 95% CI 1.43-8.25) following VPA monotherapy during pregnancy [22].

One of the most interesting meta-analyses investigating the link between VPA use and risk of major malformations is by Tanoshima *et al*. [46]. Using the cumulative meta-analysis approach, the authors detected that the risks associated with VPA and neural tube defects first became evident as early as 1992, while for other specific malformations (heart defects, oral clefts, genitourinary and musculoskeletal malformations) the cumulative risk ratios did not start to became significant until 2004 [46]. In their conventional meta-analysis, the authors detected a 2.3-fold increase in risk for overall major malformations, a 2- to 3-fold increase in risk for heart defects, oral clefts, genitourinary and musculoskeletal malformations, and an approximately 7-fold increase in risk for neural tube defects [46].

#### 2.4.1 Valproate and Neural Tube Defects

The association between maternal VPA use and neural tube defects was the very first established association in the context of VPA teratogenicity. The reports suggesting this link were immediately confirmed by the detection of a 20-fold increased risk of spina bifida in a French case-control study [47] and the further findings by another study that compared the data from several countries [48]. The absolute risk for neural tube defects following maternal VPA use has been estimated as 1-2%, which corresponds to a 10-20 fold increase in risk for the general population, and the association is particularly strong with spina bifida [35, 45].

### 2.5 Lamotrigine

The prevalences of major malformations among the studies for LTG monotherapy during pregnancy appear to be consistent. As early as 2004, the Swedish Medical Birth Registry suggested a prevalence of 4.4% in a study involving a relatively low number of pregnancies (n=90) [25]. In another study, Meador *et al*. detected a lower prevalence (1.0%) in a similar number of pregnancies [43]. International Lamotrigine Pregnancy Registry reported a prevalence of 2.2% among 1558 first trimester monotherapy exposures [49], while the analysis of the North American AED Pregnancy Registry yielded a similar prevalence of 2.0% [18]. Australian Pregnancy Register reported a prevalence of 5.2% with LTG monotherapy [50]. Although higher than in previously reported studies, this figure was similar rather than different to that of untreated women (5.2%) [50]. The recent analysis of the same register in 2019 yielded a slightly lower rate of major malformations (4.9%) following LTG monotherapy. This rate was again not significantly elevated in comparison to untreated pregnant women [14]. In a registry-based study from Denmark, the prevalence of major malformations following LTG monotherapy was detected as 3.7%, and the comparison with the control infants yielded no significant increases in the risk of major malformations (APOR 1.18; 95% CI 0.83-1) for LTG-exposed group [51].

In more recent studies, a consistently low rate of prevalences continued to emerge. The analysis of UK registry in 2014 yielded a prevalence for the major malformations as 2.3% [52], and most recently, Tomson *et al*., in the EURAP registry, reported the prevalence of major malformations after LTG use during pregnancy as 2.9% [19]. The analysis of the Health Improvement Network (THIN) data from UK yielded a rate of major malformations as 5.1% following LTG monotherapy during pregnancy, significantly higher than that of mothers without AED exposure (OR 2.01; 95% CI 1.12–3.59) [27]. Blotière *et al*.’s recent analysis of the French National Health Insurance data detected no significant associations between LTG use during the first two months of pregnancy and 23 specific malformations [24].

The pooled prevalence of major malformations for children exposed to LTG monotherapy was 2.31% in Weston *et al*.’s meta-analysis [21]. Further comparisons in the same meta-analysis yielded no significant increase in the risk of major malformations and organ-specific malformations, such as cardiac defects and neural tube malformations for the LTG-exposed children. The risk ratios for major malformations were 1.68 (95% CI 0.78 - 3.65) (compared with the children of mothers without epilepsy) and 1.07; 95% CI 0.64 - 1.77 (compared with children of the epileptic mothers using no medication during pregnancy) respectively [21]. In their recent meta-analysis, Pariente *et al*. detected similar findings, *i.e.*, no significant increase in risk for major malformations in children with in utero exposure to LTG monotherapy when compared with disease-matched (OR 1.15; 95% CI 0.62–2.16) and healthy controls (OR 1.25; 95% CI 0.89–1.74) [53]. The results from another meta-analysis by Veroniki *et al*. were also in line with the previous meta-analyses; there was no association between LTG monotherapy during pregnancy and a significant increase in risk for major (OR 0.96; 95% CI 0.72–1.25) and organ-specific malformations [22].

#### 2.5.1 Lamotrigine and Oral Clefts: The Discrepancy

Concerns exist regarding the possible association of LTG with oral clefts in infants. An earlier analysis of the EUROCAT registry by Dolk *et al*. suggested no increase in the risk of oral clefts in infants exposed to LTG monotherapy in utero [54], whereas the analysis of the North American AED Pregnancy Registry by Holmes *et al*. same year yielded a significant 10.4-fold increase (7.3 per 1000) in the risk of isolated oral clefts among the infants under the same conditions [55]. The subsequent analysis of the same North American AED Pregnancy Registry in 2012 yielded a lower rate (4.5 per 1000), which corresponds to a 6-fold increased risk [18]. Much lower rates were reported in Denmark (0.1%) [51] and EURAP registry (<1%) [19]. The latest analysis of the EUROCAT registry in 2016, which significantly extended the study population from 3.8 to 10 million births, found no significantly raised risk of oral clefts [56]. Thus, the 6-fold increase in risk of oral clefts suggested by North American AED Pregnancy Registry was not supported in other studies. Three possible reasons for this discrepancy were the differences in baseline population risk of oral clefts (1.4 per 1,000 in EUROCAT *vs* 0.7 to 1.1 per 1,000 in North American Pregnancy Registry), chance finding, or an exacerbation by confounders [56].

### 2.6 Levetiracetam

An earlier analysis by Mølgaard-Nielsen *et al*. has detected no major malformations among a small sample of Danish pregnant women (n=58) exposed to LEV monotherapy during pregnancy [51]. In North American AED Pregnancy Registry, the major malformation prevalence after maternal use of LEV monotherapy was 2.4% [18]. This number was not significantly different from that of those exposed to LTG (OR 1.2; 95% CI 0.6–2.5) [18]. The results from UK and Ireland Epilepsy and Pregnancy Register suggested an even lower prevalence (0.7%) of malformations following LEV monotherapy during pregnancy [57]. The EURAP registry provided further support for this trend, with a prevalence of 2.8% for major malformations after the use of LEV monotherapy during pregnancy [19]. The very recent analysis of the Australian Pregnancy Register detected a major malformation rate of 3.6% after maternal use of LEV as monotherapy [14]. The risk ratio (1.27; 95% CI 0.37–4.29) was not significantly different from the AED-unexposed pregnancies [14]. Blotière *et al*.’s recent analysis of the French National Health Insurance data suggested no significant associations between LEV use during the first two months of pregnancy and 23 specific malformations [24].

The earliest meta-analysis by the Motherisk team suggested a pooled major malformation rate of 2.2% following LEV monotherapy during pregnancy [58]. Later, Weston *et al*. reported a lower pooled prevalence of major malformations (1.77%) following in utero exposure LEV monotherapy in their meta-analysis [21]. For comparisons, a single study was available for LEV-exposed children *vs* children of mothers without epilepsy (North American AED Pregnancy Registry), of which the risk ratio (2.16; 95% CI 0.76–6.17) was not significantly elevated. For mothers using LEV monotherapy *vs* epileptic mothers using no medication during pregnancy, the pooled result of two studies yielded a non-significant outcome (RR 0.32; 95% CI 0.10–1.07) [21]. The authors were not unable to conduct further analyses on organ-specific malformations because no previous studies had reported data for the relevant outcomes [21]. The results from the Veroniki *et al*. study were also in agreement with Weston *et al*.; there was no association between LEV monotherapy during pregnancy with an elevated risk in major malformation rate (OR 0.72; 95% CI 0.43–1.16), and no increase in risk for organ-specific malformations was observed in the further analysis [22].

### 2.7 DISCUSSION

It is apparent that VPA monotherapy is associated with the highest rates of major malformations among all the registries presented in the details above. In contrast, LEV and LTG monotherapy have been demonstrated to have much lower rates of major malformations which is similar to that of normal populations Table (**2**). Of importance, no repeating patterns of major malformations or organ-specific malformations following LEV and LTG monotherapy during pregnancy were evident. The suggested increase in the oral clefts following LTG monotherapy was not confirmed in other studies. The major malformation rates with CBZ monotherapy seem to be higher than those of LEV and LTG but lower than those of PB and PHT.

In this context, the “newer” AEDs, LEV and LTG appear to be the safest AEDs for pregnancy for their appropriate clinical indications.

### 2.8 Dose-dependency

#### 2.8.1 Phenytoin

For PHT, a small number of studies investigated the relation between dose and risk of major congenital malformations. Samrén *et al*. reported non-significant 2.8-fold and 4.1-fold increases in risk for doses 201-300 mg/day and > 300 - 500 mg/day when compared to a dose of <200mg/day [41]. Kaneko *et al*. reported a significant association with dose yet provided no details [15]. In contrast, North American AED Pregnancy Registry and Kaaja *et al*. reported no association between PHT dose and risk of major congenital malformations [16, 18]. Similarly, there was no significant dose-response relationship for PHT and major malformations in the recent analysis of the Australian Pregnancy Register [14].

#### 2.8.2 Phenobarbital

The dose-dependent risk of major malformations for PB has been little investigated. In the EURAP registry, the prevalence was found to be dose-dependent. For ≤80 mg/day the prevalence of major malformations was lowest, at 2.7%, whereas for >80 to ≤130 mg/day and >130 mg/day, it was 6.2% and 11.7%, respectively [19]. In contrast, North American AED Pregnancy Registry found no association with dose [18].

#### 2.8.3 Carbamazepine

Using UK Register data, Campbell *et al*. also suggested a dose-dependent increase in the risk of major malformations following in utero exposure to CBZ. The authors stratified the daily dose as ≤500 mg/day, >500–≤1000 mg/day, and >1000 mg/day and calculated the major malformation rate as 1.9%, 2.7% and 5.3%, respectively. The difference was statistically significant between the doses ≤500 mg/day and >1000 mg/day (OR 2.82; 95% CI 1.20–6.64) [52]. Tomson *et al*. reported that, in doses of greater than 700 mg/day, the risk of major malformations increased significantly (7.2%) when compared to doses of less than 700 mg/day (4.5%) [19]. In contrast, no apparent dose trend for the risk of major malformations was evident in the North American Pregnancy Registry and Australian Pregnancy Register following CBZ monotherapy during pregnancy [14, 18].

#### 2.8.4 Valproate

Since the beginning of 1990s, observational studies have consistently demonstrated a dose-dependent increase in the rate of malformations following in utero exposure to VPA. The initial view of the probable safety of doses below 1000 mg/day was challenged in subsequent studies, which reported increasing VPA doses may progressively increase the risk of major malformations.

Samrén *et al*. found that the offspring of mothers who took VPA ≥ 1000 mg/day had a significant increase in risk in major malformations compared to those using <600 mg/day (RR 6.8; 95% CI 1.4-32.7) [41]. In the study by Kaneko *et al*., VPA alone showed a significant correlation between dose and rate of major malformations [15]. Almost 90% of VPA-exposed children with malformations were exposed to a dose ≥ 1000 mg/day [15]. Artama *et al*., in their analysis of the Finnish Birth Registry, detected a major malformation rate of 9.5% for doses ≤1,500 mg/day and 23.8% for doses >1,500 mg/day, respectively [42]. Similarly, Meador *et al*. found that VPA was the only AED associated with dose-dependent effects leading to serious adverse fetal outcomes [43]. The rate of serious adverse outcomes was detected as 24.2% *vs.* 9.1% for doses > and < 900 mg/day, respectively [43].

The earlier analysis of UK Epilepsy and Pregnancy Register suggested increasing, although not significant, risk of major malformations with increasing doses of VPA. For ≤600 mg/day the prevalence of major malformations was lowest, at 4.1%, whereas for >600 to ≤1000 mg/day and >1000 mg/day, it increased to 6.1% and 9.1%, respectively [17]. Diav-Citrin *et al*. reported an 8-fold increase in the rate of major malformations for VPA exposure of over 1000 mg/day (RR 8.72; 95% CI 4.16-18.30) compared with the control group [44]. Interestingly, daily VPA doses <1000 mg/day were not associated with an increased risk of major malformations [44]. North American AED Pregnancy Registry yielded similar results [18]. In pregnancies resulting in malformations, the median average dose of VPA was 1000 mg/day, whereas in pregnancies without malformations, it was 750 mg/day [18]. Tomson *et al*. have investigated the dose-dependent risks of VPA in mono and polytherapies in the EURAP registry and compared VPA monotherapy with VPA + LTG and VPA +another AED (excluding LTG) [59]. In all three groups, the rate of major malformations was observed to increase with increasing doses of VPA. For the lowest VPA doses (<700 mg/day) the rates of major malformations were 5.9% for monotherapy, 7.0% for VPA + LTG, and 5.4% for VPA + another AED. For the highest doses (≥1,500 mg/day), the rates were detected as 24.0% for monotherapy, 31% for VPA + LTG and 19.2% for VPA + another AED [59]. In the recent results of EURAP registry, dose-dependent malformation risks with VPA were also evident [19]. The authors stratified the daily dose as ≤650 mg/day, >650–≤1450 mg/day and >1450 mg/day, and detected the respective major malformation rates as 6.3%, 11.3% and 25% [19]. The latest analysis of the Australian Pregnancy Register showed that the malformation hazard increases after doses of 600 mg/day and becomes statistically significant at 700 mg/day [14].

#### 2.8.5 Lamotrigine

An earlier study that analyzed UK Epilepsy and Pregnancy Register by Morrow *et al*. detected that the mean daily dose of LTG was significantly higher for cases with a major malformation than for cases without (352.4 mg *vs* 250.6 mg; p=0.005) [17]. Further analysis suggested a cut-off value of 200 mg. The prevalence of major malformations were 1.3% and 1.9% for doses <100 mg/day and 100 to 200 mg/day, rising to 5.4% for doses over 200 mg/day [17]. Later in 2014, using UK and Ireland Epilepsy and Pregnancy Registers data, Campbell *et al*. detected no significant difference in the mean daily dose for cases with or without for LTG (283.7 mg *vs* 255.4 mg p=0.20). They observed a non-significant trend for an increase in the rate of malformations with LTG monotherapy with increasing total daily dose. The rate of malformations was 2.1%, 2.4% and 3.4% for doses 0–≤200 mg/day, >200–≤400 mg/day, and >400 mg/day, respectively [52]. In the EURAP study, the prevalence of major congenital malformations was 2.5% *vs* 4.3% for doses ≤325 mg/day and >325 mg/day, respectively. This difference was statistically significant [19].

Contrary to these findings, International Lamotrigine Pregnancy Registry’s final results suggested no dose-response relationship; however, the authors pointed out that the number of exposures over 600 mg/day was limited [49]. Similarly, North American AED Pregnancy Registry and Australian Pregnancy Register failed to find an association between LTG dose during pregnancy and major malformations [14, 18, 60]. The analysis of the Danish Medical Birth Registry also detected no dose-response effect of LTG use on the risk of any major malformations (≤250 mg/day *vs* > 250 mg/d) [51].

#### 2.8.6 Levetiracetam

The analysis of the North American AED Pregnancy Registry reported no dose-dependent increase in the risk of major malformations for LEV therapy during pregnancy [18]. Similarly, no apparent dose trend for maternal LEV monotherapy or major congenital malformations was observed in either UK or Ireland Epilepsy and Pregnancy Registers [57]. Consistent with these findings, no dose-dependent increases in the rate of the major congenital malformations were evident for LEV in the EURAP registry [19] and, most recently, the Australian Pregnancy Register of Antiepileptic Drugs [14].

#### 2.8.7 DISCUSSION

For VPA, dose-dependency with the major malformations can be considered as proven, however more studies are needed to define the thresholds Table (**3**). Currently <600 mg/day seems to be relatively safer for VPA. For PHT, PB, CBZ, and LTG, some studies showed a dose-dependent increase in malformation risks, however these were not confirmed in other important studies. This also needs to be further studied, however for the time being, these AEDs should be administered at the lowest effective dose which ensures optimal seizure control for pregnant women. Interestingly, no single study to date that we are aware of reported a dose-dependent increase in risk of major malformations for LEV.

### 2.9 Mono *vs* Polytherapy

The findings to date point to VPA as one of the agents which may increase the malformation rate when included in polytherapies. In the earlier analysis of the International Lamotrigine Pregnancy Registry, the major malformation rate was observed to be much higher (12.5%) in pregnancies with exposure to LTG polytherapy, including VPA compared to pregnancies with exposure to LTG polytherapy excluding VPA (2.7%) [61]. In the analysis of UK Epilepsy and Pregnancy Register, Morrow *et al*. showed that the combinations with VPA carried a significantly higher risk of major malformations than those without (OR 2.49; 95% CI 1.31–4.70) [17]. The Finnish cohort study by Artama *et al*. detected that maternal AED polytherapy with VPA during pregnancy significantly increased the risk of major malformations (OR 3.54; 95% CI 1.42–8.11) whereas no significant increases in risk were evident following AED polytherapy without VPA [42]. Later, similar findings were reported from North American AED Pregnancy Registry [62]. In this study, risks of major malformations in women taking CBZ and LTG as polytherapy in the first four months of pregnancy were compared with those taking these drugs as monotherapy, and with the two unexposed control groups. Both CBZ + VPA (OR 6.2; 95% CI 2.0–16.5) and LTG + VPA (OR 5.0; 95% CI 1.5–14.0) groups exhibited significantly higher risk of major malformations than the CBZ+ any other AEDs (OR 0.8; 95% CI 0.3–1.9) and LTG+ any other AEDs (OR 1.5; 95% CI 0.7–3.0) (Holmes 2011). The latest analysis of the International Lamotrigine Pregnancy Registry in 2015 found results similar to the previous study, *i.e.*, the malformation rate following LTG polytherapy including VPA during pregnancy appeared to be much higher (10.7%) than those exposed to LTG polytherapy excluding VPA (2.8%) [61]. In an analysis of EURAP registry, Tomson *et al*. reported that the risk of major malformations associated with polytherapy is primarily influenced by VPA and found an interesting trend towards a lower rate of malformations in the VPA + other AEDs group (19.2%) compared to VPA monotherapy (24.0%) for the highest VPA doses (≥1,500 mg/day) [59].

Another AED suspected of increasing the risk of major malformations when used in polytherapy is topiramate. Keni *et al*., using the Kerala Registry of Epilepsy and Pregnancy (KREP) data in India, investigated the rate and pattern of malformations in AED monotherapy *vs* dual therapy. Among the AED dual therapies evaluated, the rate of major malformations appeared to be highest for the combinations which included topiramate (OR 14.6; 95% CI 1.88–113.83), primidone (OR 12.6; 95% CI 1.35–119) and VPA (OR 5.43; 95% CI 0.72–40.81) [63]. Recent findings from Australian Pregnancy Register suggested that the major malformation rates in polytherapy remained high despite a decline in VPA use. The authors highlighted the increase in the use of topiramate in this period and detected that topiramate in polytherapy, like VPA, is associated with an increased rate of major malformations [14].

### 2.10 DISCUSSION

Until the last decade, the combination of two or more AEDs was considered to carry a greater major malformation risk than using AEDs as a monotherapy. It was therefore recommended that women of childbearing age with epilepsy should avoid polytherapy whenever possible. However, this approach could be problematic in the clinical setting since, for some cases, monotherapy may mean reduced seizure control. Several studies challenged this view, proposing that rather than polytherapy itself, particular drugs in polytherapy such as VPA or topiramate contributed to this increased risk [60, 64]. Despite these findings, this area needs considerably more research in order to reach conclusions for a number of polytherapy combinations, taking into account different doses and baseline factors [65-68].

## 3 PHARMACOKINETICS DURING PREGNANCY AND CLINICAL IMPLICATIONS

### 3.3 Carbamazepine

CBZ is 75% protein bound and metabolized by CYP3A4, CYP1A2 and CYP2C8 enzymes [69]. The increases in the free fraction were found to be variable among the studies (101%-116%) [70], and the decrease in the total concentrations was not as high as PHT or PB (drops to 79%) when compared to non-pregnant women [70]. Johnson *et al*., for instance, found no significant change in the total and free CBZ, and in total and free CBZ-epoxide clearances during pregnancy [71]. Another important finding of this study was that seizure frequency worsening was not associated with decreased concentrations of total or free CBZ during pregnancy [71]. Reisinger *et al*. showed that pregnant women on CBZ treatment needed fewer dose adjustments compared to LTG and LEV during pregnancy. These pregnant women were also shown to have a lower rate of worsened seizure control (0%) when compared to LTG, LEV, and other mono and polytherapies. However, one limitation of this study was the low number (n=6) of pregnant women in the CBZ group [68]. Similar to these findings, Battino *et al*. also reported that worsening seizure control and the mean dose increase were less common among pregnant women on CBZ therapy [72]. In addition, the pregnant women initially on CBZ monotherapy less frequently needed an additional AED between the first and the third trimesters [72].

### 3.4 Valproate

VPA is another AED that is highly bound to proteins (87-95%) and metabolized by UGT1A3, 2B7 and CYP2C9, CYP2A6 and CYP2B6 [69, 73]. Although total plasma drug concentrations appear to be decreased during pregnancy (23-40%), no significant change in the free fractions was evident [69, 74]. Remarkably, there appears to be a complete absence of data on how pregnancy affects VPA clearance [69, 75].

### 3.5 Lamotrigine

The serum protein binding of LTG is relatively lower (55%) and it is metabolized by glucuronosyltransferase enzymes UGT, UGT1A4, and 2B7 [69]. No reports regarding the change in the free fraction were found among the studies [70], however, a substantial increase in the clearance (212%) was evident among the pregnant women on LTG therapy in the third trimester [70]. This increase appears to start as early the 5^th^ gestational week [76] and resumes to pre-pregnancy values within the first few weeks postpartum [75]. It is interesting that some studies indicated that the change may vary between individuals [77, 78]. A population-based model showed that the varying degrees of change (10-fold between groups) in the LTG clearance was significantly affected by gestational age, and that therapeutic drug monitoring may be needed to optimize the therapy [79]. A meta-analysis also suggested that the monitoring of LTG concentrations reduces the rate of seizure worsening (0.30 95% CI 0.21-0.^41^]) when compared to the exclusive clinical feature monitoring (0.73 95% CI 0.56-0.86) [80].

### 3.6 Levetiracetam

LEV is pharmacokinetically different from the AEDs above since it is not bound to serum proteins. The main route of elimination is renal; however, as much as 30% occurs through hydrolysis [69]. Similar to LTG, its clearance in pregnant women was reported to significantly increase (269%), particularly during the 3^rd^ trimester, when compared to the non-pregnant women [70]. However, this view is challenged in recent studies, which suggest that the main increase in the clearance of LEV occurs during the first trimester [81, 82]. The data on seizure worsening are limited for LEV, however, one recent study showed that a lower ratio to target concentrations (<0.65) was associated with seizure worsening [81], which implies potential benefits for therapeutic drug monitoring during pregnancy.

### 3.7 DISCUSSION

The need for therapeutic drug monitoring (TDM) during pregnancy varies among these AEDs. LEV and LTG appear to require a closer clinical observation, dosage adjustments and therapeutic drug monitoring, whereas CBZ appears to be associated with less dosage adjustments and therefore, might be beneficial for the treatment of pregnant women with relevant indications and with limited access to TDM [74]. Further studies are definitely necessary to explore how pregnancy affects VPA clearance.

## 4 CONCLUSION FOR PREGNANCY

The main question that the clinical teratologist faces in daily practice is whether a medication increases the risk of overall major or specific malformations, and if so, by how much. However, the teratogenic risk is not the only significant parameter that affects the choice of an AED; others are seizure type and severity, prior response to the treatment, and comorbidities of the patient. Therefore, it should be highlighted that there is no single approach that can be applied equally to all patients. The decision should be individualised through a mutual understanding between the patient and the physician, considering not only evidence, but also the patients’ expectations and teratogenic risk perception. Open and effective communication between patient and physician is important in finding the delicate balance between providing effective maternal seizure control, and minimising the possible fetal adverse effects of the disease and AEDs. The considerations regarding the use of AEDs during pregnancy is summarised in Table **4** as highlights.

## 5 BREAST FEEDING

Parameters considered to be important in calculating and interpreting the infant’s exposure to medications *via* human milk are the milk/plasma ratio, relative infant dose, infant plasma concentration and the ratio of infant plasma concentration to the maternal plasma concentration, which are discussed elsewhere [83]. From the clinical teratologist’s perspective, the two key parameters in assessing the potential risks of medication use during breastfeeding are the relative infant dose, and the adverse effect data from the previous case series and reports. Infant plasma concentration and the ratio of the infant plasma concentration to the maternal concentration are the other important parameters [84]. Infant plasma concentration to the maternal concentration can be advantageous in minimizing the differences (*e.g.* clearances) between the infant and the mother. It may be particularly beneficial in estimating the breastfeeding compatibility of medicines with long-half lives, for which the fluctuations in plasma levels may act as a confounder [84].

Relative Infant Dose (RID) is calculated as the dose that the infant is exposed to *via* milk divided by the weight-adjusted dose of the mother. Infant exposure is considered as minimal when RID is below 2%, small when between 2 and 5%, moderate when 5-10% and high when above 10% [85]. Breastfeeding is usually considered as safe if the RID <10% [86], although there are rare exceptions where adverse effects in the infants were reported with RID <10% (*e.g.* aripiprazole). Similarly, for some medications, an RID above 10% does not necessarily contraindicate breastfeeding (*e.g.* fluconazole).

It is also important to note that the combination therapy with AEDs is frequent, and that particular AEDs can induce (*e.g.* PHT, CBZ) or inhibit (*e.g.* VPA) the metabolism of others [87]. Therefore, the calculated RID should be interpreted carefully.

### 5.1 Phenytoin

A review of the cases in LactMed, which report the concentrations in milk samples of several women who used PHT during breastfeeding (90 to 1000 mg/day) suggested an RID between 0.5 to 8% [88], which is compatible with breastfeeding [89].

The great majority of anecdotal cases reporting the assessment of the adverse effects in infants exposed to PHT *via* breastmilk suggested no severe or serious adverse effects [88]. However, there are a few notable cases. A 5-day old infant whose mother was on PHT and PB therapy, experienced a serious methemoglobinemia and sedation, which was resolved with the discontinuation of breastfeeding (dechallenge) and then aggravated upon its reintroduction (rechallenge) [88]. Drowsiness and feeding difficulties were also reported in a small number of anecdotal cases in which mothers were using PHT with other AEDs or drugs (*e.g.* CBZ, clemastine *etc.*) [88, 90]. Slower weight gain was notified among the infants of mothers using various AEDs compared to the infants of the epileptic mothers using no AEDs [91]. PHT is classified among the drugs which are compatible with breastfeeding by the American Academy of Pediatrics [92].

### 5.2 Phenobarbital

Hale reported an RID of 24% [86] for PB. The infant plasma concentrations were reported to change between 2.0 to 54.7μg/mL [89]. Accumulation of the drug is possible because the elimination capacity is reduced in neonates. Several cases of drowsiness were reported in infants whose mothers were using PB during breastfeeding [93]. It is interesting to note suggestions that withdrawal symptoms in infants exposed to PB during pregnancy are decreased by PB exposure through breastmilk [93]. Given the high RID and relatively higher frequency of the adverse effects in infants, The American Academy of Pediatrics has classified PB as a drug to be used with caution during breastfeeding [92].

### 5.3 Carbamazepine

The reported RID of CBZ changes between 3.8 and 5.9% by Hale [86]. Infant plasma concentrations were reported to vary between <0.5 ng/L and 4.7 μg/mL [89]. However, accumulation of the drug is possible. In most infants exposed to CBZ through breastmilk, no serious or severe adverse effects were reported [94]. However, sedation, feeding difficulty due to poor sucking and withdrawal reactions were detected in some cases where CBZ was used as mono or polytherapy [94]. Although some of these findings were attributable to concurrent therapy or other drugs taken, the effect of CBZ cannot be ruled out in these reports [94]. Cholestatic hepatitis, a known adverse effect of CBZ, was reported in a 3-week-old breastfed infant whose mother was on CBZ monotherapy both during pregnancy and breastfeeding [95]. The infant was admitted to the hospital because of persistent jaundice, and after cessation of breastfeeding, parameters of cholestasis (conjugated bilirubin, GGT, alkaline phosphatase) gradually returned normal [95]. Of note, the serum transaminases increased to a transient peak 6.5 weeks after discontinuation of breastfeeding [95]. Interestingly, two other published reports associated CBZ therapy during breastfeeding with liver dysfunction in the breastfed infants [96, 97]. As suggested by Frey *et al*., there were similarities in all three infants: 1) all were breastfed; 2) all mothers were taking CBZ during pregnancy and breastfeeding (400 - 600 mg/day); 3) all had a transient increase in liver enzymes 4) other causes of hepatitis were ruled out [96]. The American Academy of Pediatrics considers CBZ as probably safe during breastfeeding [92].

### 5.4 Valproate

The reported RID of VPA is between 0.99% to 5.6% and compatible with breastfeeding [86] and infant plasma concentrations are reported to vary between undetectable levels to 13.4 μg/mL [89]. The majority of the reported cases in LactMed suggest no serious adverse reactions in infants exposed to VPA through breastmilk [98]. Sedation occurred in one infant whose mother was taking VPA with primidone; the latter was thought to be responsible, although the former might also have contributed to the adverse effect by inhibiting primidone metabolism and increasing its levels. Sedation was reported to respond to the discontinuation of breastfeeding in this infant [98].

An infant with thrombocytopenic purpura, anemia, and reticulocytosis whose mother was using VPA monotherapy during breastfeeding was reported by Stahl *et al*. [99]. The clinical and laboratory parameters returned to normal within 2.5 months following the discontinuation of breastfeeding. Stahl *et al*. proposed a causality with VPA, but others found this association questionable [100]. The American Academy of Pediatrics classifies VPA as compatible with breastfeeding [92].

### 5.5 Lamotrigine

LTG has a RID between 9.2% to 18.3% [86] and its infant plasma concentrations reported to vary between <0.1 and 12.7 mg/mL [89]. Most infants breastfed while their mothers were using LTG were reported to be free of adverse effects [101]. However, a probable association between LTG and an apneic episode necessitating cardiac compressions was described in a neonate [102]. Sedation, hypotonia, weight loss and liver damage were the adverse effects reported to the French Pharmacovigilance Database in breastfed children whose mothers were on LTG therapy [103]. In addition, rashes were reported in a few cases [101]. Elevated platelet counts with no additional adverse effects were also observed [101]. LTG is classified by the American Academy of Pediatrics as a drug with unknown effects that may be of concern [92].

### 5.6 Levetiracetam

Reported RID of LEV changes between 3.4-7.8% [86] which is in the compatible range for breastfeeding. The reported infant plasma concentrations change between 4 and 20 μmol/L [89]. No adverse effects were reported in the majority of the infants exposed to LEV through breastfeeding [104]. However, sedation, hypotonicity and feeding difficulties, which improved after discontinuation of breastfeeding, were reported in some infants whose mothers were on LEV therapy with other AEDs such as PHT, VPA, clobazam and lacosamide [104-107]. Following the cessation of breastfeeding due to the concerns of adverse effects, one infant whose mother was using primidone/PHT with LEV experienced withdrawal seizures, which disappeared after breastfeeding resumed [108].

### 5.7 Developmental Outcomes During Breastfeeding

For years, developmental outcomes following AED exposure during breastfeeding remained as an understudied domain [109]. This scene has changed in the last decade with the valuable evidence provided by important studies [110-112]. The earlier report from The Neurodevelopmental Effects of Antiepileptic Drugs Study (NEAD) showed no adverse cognitive outcomes associated with AED exposure *via* breastfeeding in children at 3 years of age compared with non-breastfed children [110]. The follow-up report at the age of 6 years, in smaller sample size, again demonstrated reassuring results [112].

Moreover, breastfed children were found to have significantly higher overall and verbal IQ. This result should be cautiously interpreted since there are inherent limitations associated with the study design. Nevertheless, it may also suggest that the possible negative IQ impact as a result of the prenatal exposure to some AEDs, such as VPA, may recover to some degree, possibly with breastfeeding [109]. The Norwegian Mother and Child Cohort Study (MoBa) reported similar findings [111]. There were no deleterious effects of breastfeeding on development at ages 6 to 36 months in children of mothers using AEDs. The authors also observed that continuous breastfeeding demonstrated a more favorable outcome, particularly for early autistic traits and was also associated with a lower risk of poor weight gain during the postnatal period [111]. The considerations regarding the use of AEDs during breastfeeding is summarised in Table **5**.

## Figures and Tables

**Table 1:** The characteristics of three largest registry-based studies which assess the risk of major congenital malformations following prenatal AED exposure

-	**Hernandez-Diaz *et al.*, 2012 (NAAPR)**	**Campbell *et al.*, 2014 (UKEPR)**	**Tomson *et al.*, 2018 (EURAP)**
**Country**	North America	United Kingdom (UK)	42 countries with more than 1500 collaborators
**Study Period**	1997-2011	1996 - 2012	1999 - 2016
**Design/setting**	Registry-based cohort	Registry-based cohort	Longitudinal, prospective cohort study
**Data source**	The North American AED (Antiepileptic Drug) Pregnancy Registry	UK and Ireland Epilepsy and Pregnancy Registers	EURAP international registry
**Number of participants**	5,667 AED-exposed and 442 AED unexposed women / 5,265 infants*(calculated from tables)	5,206 pregnant women / 4,975 infants*(calculated from tables)	7,355 pregnancies / 7,255 infants
**Number of events (number of pregnancies/ number of major malformations)**	Lamotrigine (n=1562/31) Carbamazepine (n=1033/31) Phenytoin (n=416/12) Levetiracetam (n=450/11) Valproate (n=323/30) Phenobarbital (n=199/11)	Valproate (n=1220/82) Carbamazepine (n=1657/43) Lamotrigine (n= 2098/49)	Lamotrigine (n=2514/74) Carbamazepine (n=1957/107) Phenytoin (n=125/8) Levetiracetam (n=599/17) Valproate (n=1381/142) Phenobarbital (n=294/19)
**Unexposed: **	Total births: 442	N/A	N/A
**Inclusion criteria**	-All women with AED were at least 7 months’ gestation, and postpartum. All participants must have been pregnant and they must have used AED at some time during her pregnancy.	All pregnancies who referred to the UKEPR exposed to valproate, carbamazepine or lamotrigine in monotherapy in the first trimester between December 1996 and December 2012.	All pregnancies which exposed to AEDs and enroled in EURAP within gestation week 16 were included. The pregnant women with unknown outcome were also included for prospective assessment.
**Exclusion criteria**	N/A	Antenatal diagnosis of a probable or definite MCM are excluded.	-Retrospectively identified pregnancies, -Those occurring in women without epilepsy, -Those for which physicians did not submit reports within preset deadlines, -Those for which follow­up was not yet completed at the current census, -Those antiepileptic drugs were switched or withdrawn during the first trimester, -Those exposed to antiepileptic drug combination therapy or to other potentially teratogenic drugs, -Those with comorbidities associated with teratogenic risks -Spontaneous abortions, -Abortions induced for causes other than fetal abnormalities, -Pregnancies in which fetal outcome could not be determined, -Pregnancies that resulted in offspring with genetic or chromosomal abnormalities.
**Exposure**	Lamotrigine, Carbamazepine, Levetiracetam, Phenytoin, Topiramate, Valproate, Phenobarbital, Oxcarbazepine, Gabapentin, Zonisamide, Clonazepam	Valproate, Carbamazepine, Lamotrigine	Valproate, Phenobarbital, Phenytoin, Carbamazepine, Topiramate, Oxcarbazepine, Lamotrigine, Levetiracetam.
**Exposure time window**	During the first 4 lunar months after the last menstrual period	During the first trimester	During the first trimester
**Control **	The reference groups of the study were women exposed to lamotrigine or not taking an AED and without epilepsy.	N/A	The data comparisons were made with different doses of AED’s or other AED’s doses’ or lamotrigine.
**Method of congenital malformation diagnosis**	Computer-assisted interviews with mothers and then for verification of baby’s health were asked their physicians with letter. A major malformation was defined as a structural abnormality with surgical, medical, or cosmetic importance.	According to EUROCAT registry	MCM's were characterized as structural malformations with surgical, medical, functional, or cosmetic importance, and categorized according to the 2005 EUROCAT criteria
**Covariates for adjustment**	Start and stop dates of each AED taken, dose, frequency, changes in medication, indication, and, if epilepsy, number and type of seizures during pregnancy; demographic characteristics (age, mother’s education, married, ethnicity of mother/father (Caucasian), primiparity, folic acid supplement, cigarette smoking, alcohol status, indication of epilepsy, age first seizure, seizures during pregnancy); habits, such as cigarette smoking, alcohol intake, and use of illicit drugs; medical conditions; use of other medications; family history; and results of any prenatal testing	Maternal age at delivery, number of previous pregnancies, folic acid consumption, family history for MCM	Maternal age at time of conception, pregnancy at time of enrolment, parent history of major congenital malformations, geographical region, parity, type of epilepsy, generalized tonic-clonic seizures during first trimester, educational level of the father or mother, folic acid intake, sex of the child

**Abbreviations** are as follows: Not available (N/A), major congenital malformation (MCM)

**Table 2:** Major malformation percentages in various pregnancy registries.

-	**Hernandez-Diaz, 2012 (NAAPR)^a^**	**Campbell, 2014 (UKEPR)^b^**	**Tomson, 2018 (EURAP)^c^**	**Blotière 2019 (French** **National Health Insurance Claims Data)^d^**	**Vajda 2019 (Australian Pregnancy** **Register (APR))^e^**
Phenytoin (PHT)	2.9% (95% CI 1.5–5.0) n=12/416	NA	6.4% (95% CI 2.8–12.2) n=8/125	NA	2.3% n=1/44
Phenobarbital (PB)	5.5% (95% CI 2.8–9.7) n=11/199	NA	6.5% (95% CI 4.2–9.9) n=19/294	2.5% n=2/80	NA
Carbamazepine (CBZ)	3.0% (95% CI 2.1–4.2) n= 31/1033	2.6% (95% CI 1.9%-3.5%) n=43/1657	5.5% (95% CI 4.5–6.6) n=107/1957	1.2% n=6/512	5.9% n=24/409
Valproate (VPA)	9.3% (95% CI 6.4–13.0) n=30/323	6.7% (95% CI 5.5%-8.3%) n=82/1220	10.3% (95% CI 8.8–12.0) n=142/1381	6.0% n=55/913	14.8% n=43/290
Lamotrigine (LTG)	2.0% (95% CI 1.4–2.8) n=31/1562	2.3% (95% CI 1.8%-3.1%) n=49/2098	2.9% (95% CI 2.3–3.7) n=74/2514	1.5% n=44/2997	4.9% n=20/406
Levetiracetam (LEV)	2.4% (95% CI 1.2–4.3) n=11/450	NA	2.8% (95% CI 1.7–4.5) n=17/599	1.2% n=7/579	3.6% n=5/139

^a,b,c^ All data were given as percentage, 95% confidence interval of prevalence, and the number of malformations among pregnancies exposed to the particular AED.

^d, e^ All data were given as percentage, and the number of malformations among pregnancies exposed to the particular AED.

^d^ Percentages were calculated from the raw data in the article.

^d,e^ 95% Confidence interval (CI) was not reported.

**Table 3:** Frequency of major malformations among different dose categories of AEDs.

**AEDs &** **Studies**	**Dose** **(mg/day)**	**Number of pregnancies exposed**	**Number of congenital malformations**	**Prevalence/ Percentage of congenital malformations**	**Confidence interval**	**P value**
**Phenytoin**	-	-	-	-	-	-
Kaneko, 1999 (Japan, Italy and Canada)	50≤ to <100	8	0	0.0^a^	NA	0.220
	100≤ to <200	21	1	4.8^a^	NA	
-	200≤ to <300	60	5	8.3^a^	NA	
-	300≤ to <400	37	5	13.5^a^	NA	
-	400≤	6	1	16.7^a^	NA	
**Phenobarbital**	-	-	-	-	-	-
Tomson, 2018 (EURAP)	≤80	73	2	2.7	0.3–9.5	0.0390
	80< to ≤130	161	10	6.2	3.0–11.1	--
	130<	60	7	11.7	4.8–22.6	--
		-	-	-	-	-
Kaneko, 1999 (Japan, Italy and Canada)	≤50	6	1	16.7^a^	NA	0.120
	50≤ to <100	14	1	7.1^a^	NA	
	100≤ to <150	28	2	7.1^a^	NA	
	150≤ to <300	29	0	0.0^a^	NA	
	300≤	2	0	0.0^a^	NA	
**Carbamazepine**	-	-	-	-	-	-
Tomson, 2018 (EURAP)	≤700	1276	58	4.5	3.5–5.8	0.0140
	>700	681	49	7.2	5.4–9.4	--
		-	-	-	-	-
Campbell, 2014 (UKEPR)	≤500	721	14	1.9	1.2–3.2	-
	500< to ≤1000	739	20	2.7	1.8–4.1	0.33^b^
	1000<	170	9	5.3	2.7–9.5	0.01^b^
Morrow, 2006 (UKEPR)	<400	401	7	1.7	0.8–3.6	-
	400 to 1000	385	10	2.6	1.4–4.7	-
	1000<	92	3	3.3	1.1–9.2	-
Kaneko, 1999 (Japan, Italy and Canada)	200≤ to <400	25	1	4.0^a^	NA	0.673
	400≤ to <600	23	2	8.7^a^	NA	
-	600≤ to <800	45	2	4.4^a^	NA	
-	800≤ to <1000	35	2	5.7^a^	NA	
-	1000≤	30	2	6.7^a^	NA	
**Valproate**	-	-	-	-	-	-
Tomson, 2018 (EURAP)	≤650	600	38	6.3	4.5–8.6	<0.0001
	650< to ≤1450	666	75	11.3	9.0–13.9	--
	1450<	115	29	25.2	17.6–34.2	--
		-	-	-	-	-
Tomson, 2015 (EURAP)	<700	648	39	6.0	4.4–8.1	<0.0001
	700≤ to <1500	755	81	10.7	8.7–13.1	
	1500≤	185	44	23.8	18.2–30.4	
		-	-	-	-	-
Campbell, 2014 (UKEPR)	<600	476	24	5.0	3.4–7.4	-
	600≤ to <1000	426	26	6.1	4.2–8.8	0.49^b^
	1000≤	297	31	10.4	7.4–14.4	0.0045^b^
Hernandez-Diaz, 2012 (NAAPR)	1≤ to <500	NA	NA	Under 5%^e^	NA	NA
	501≤ to <999	NA	NA	Between 5-10%^e^	NA	NA
	1000≤ to ≤1500	NA	NA	Between 10-15%^e^	NA	NA
-	1500<	NA	NA	Above 25%^e^	NA	NA
Diav-Citrin, 2008 (Israeli Teratogen Information Service)	<1000	78	1	1.3	NA	0.001^b^
	1000≤	32	7	21.9	NA	
Morrow, 2006 (UKEPR)	<600	266	11	4.1	2.3–7.3	-
	600 to 1000	247	15	6.1	3.7–9.8	-
	1000<	186	17	9.1	5.8–14.1	-
Artama, 2005 (Finnish Medical Birth Registry)	≤1500	NA	23 (95/1000 prevalence)		OR 3.68 (1.97–6.86)^ d^	
	1500<	NA	5 (238/1000 prevalence)		OR 10.89 (2.90–34.3)^ d^	
Kaneko, 1999 (Japan, Italy and Canada)	<600	19	0	0.0^a^	NA	0.004
	600≤ to <800	19	1	5.3^a^	NA	
-	800≤ to <1000	16	0	0.0^a^	NA	
-	1000≤	17	8	47.1^a^	NA	
**Lamotrigine**	-	-	-	-	-	-
Tomson, 2018 (EURAP)	≤325	1870	46	2.5	1.8–3.3	0.0145
	325<	644	28	4.3	2.9–6.2	--
		-	-	-	-	-
Campbell, 2014 (UKEPR)	≤200	1143	24	2.1	1.4–3.1	-
	200< to ≤400	665	16	2.4	1.5–4.0	0.67^b^
	400<	267	9	3.4	1.9–6.5	0.22^b^
Cunnington, 2011(GSK Pregnancy Registries)	>0–100	276	7^c^	2,5	NA	NA
	101–200	556	9^c^	1,6	NA	NA
	201–300	274	10^c^	3,6	NA	NA
	301–400	220	3^c^	1,4	NA	NA
	401–600	153	5^c^	3,3	NA	NA
	601–1,200	44	7^c^	0,0	NA	NA
Molgaard-Nielsen, 2011 (Danish Medical Birth Registry)	≤250	766	31	4.0	adjOR 1.29 (0.88-1.90) ^d^	
	250<	253	7	2.8	adjOR 0.84 (0.39-1.82) ^d^	
Morrow, 2006 (UKEPR)	<100	151	2	1.3	0.4–4.7	-
	100 to 200	208	4	1.9	0.8–4.8	-
	200<	279	15	5.4	3.3–8.7	-

^a^ Percentages were calculated from the raw data in the article.

^b^ Compared with the lowest dose range

^c^ Number of congenital malformations were calculated from the raw data in the article.

^d^ Comparisons were done using the unexposed group.

^g^ These data were extracted from the figure in the respective article.

**Table 4:** A summary of the considerations regarding AED use during pregnancy.

**Major Malformations**
• Maternal VPA use has the highest risk of major malformations and should be avoided if possible. When it is the only choice, the dose should be minimized (<600 mg/day) while ensuring optimum seizure control.
• The risk of major malformations following PHE and PB use during pregnancy seems to be higher than LTG, LEV, and CBZ but lower than VPA.
• CBZ is associated with an intermediate risk for major malformations, which appears to be lower than those of PHE and PB.
• For LTG and LEV, consistent data indicates no or minimal risk for structural malformations when used during pregnancy.
**Organ-specific Malformations**
• CBZ may increase the odds of offspring having a spina bifida by 2 to 10 fold, which approximates an absolute risk of 0.2-1%, considering the prevalence of this malformation.
• The absolute risk of spina bifida risk in the offspring following VPA use is higher (1-2%) than that of CBZ. Maternal VPA use has also been associated with orofacial clefts, heart defects and hypospadias.
• PB has been associated with cardiac malformations.
• Some studies suggested an increased risk of oral clefts with LTG monotherapy, but this is not confirmed in other studies.
**Dose-dependent Risk of Major Malformations**
• VPA has been consistently shown to pose a dose-dependent risk of major malformations and therefore its dose should be minimized (<600 mg/day).
• Although some studies have shown a dose-dependent increase in the risk of major malformations for LTG, CBZ, PHT and PB, other studies failed to show such an association.
• Interestingly, for LEV, no study showed a dose-dependent increase in the rates of major malformations.
• Although current evidence suggests no consistent dose-dependent increase in the rate of major malformations with PHT, PB, CBZ, LTG, and LEV, they should be administered at the lowest effective dose which ensures optimal seizure control for pregnant women.
**Polytherapy *vs* Monotherapy**
• Current evidence points to VPA and topiramate as the agents more likely to increase the malformation rate when included in polytherapies.
**Pharmacokinetics and Clinical Implications**
• Compared to CBZ, PHT, and PB, a higher increase in the clearance of LTG and LEV during pregnancy is apparent, and therefore these two may necessitate a closer clinical follow-up, therapeutic drug monitoring and dosage adjustments.
• Therapeutic drug monitoring seems to be less important for CBZ. CBZ is associated with less dose adjustment and seizure worsening, and therefore might be advantageous in pregnant women with focal-onset seizures whose access to therapeutic monitoring services is limited.
• There appears to be a complete absence of data on how pregnancy affects VPA clearance and further studies are needed.

**Table 5:** Highlights regarding AED use during breastfeeding.

• None of these drugs are contraindicated during breastfeeding, although the reported RID of LTG and PB can far exceed the threshold that is assumed to be safe (10%).
• A few well-designed studies showed no association between AED exposure *via* breastfeeding and adverse cognitive outcomes in children. Of interest, favourable trends on the outcomes were reported. More studies are needed in this area, particularly focusing on new AEDs such as levetiracetam.
• Sedation appears to be more common with PB or combinations in which PB is also used.
• Withdrawal symptoms after abrupt discontinuation of breastfeeding were experienced for PB and LEV (PB was a concomitant drug among the cases).
• A repeating pattern of liver dysfunction was detected for CBZ. The clinician may consider monitoring liver enzymes of infants exposed to CBZ *via* breast milk.
• LTG was reported with a wider spectrum of serious adverse events compared to other drugs.
• Rare but serious adverse events have been reported, and therefore, the clinician and the mother should be vigilant.
• Infants whose mothers use these drugs as mono or polytherapy during breastfeeding should be closely monitored with regard to drowsiness, sedation, irritability, feeding difficulties and age-appropriate growth.
